# Reversing B cell aging

**DOI:** 10.18632/aging.100313

**Published:** 2011-03-28

**Authors:** Ramit Mehr, Doron Melamed

**Affiliations:** ^1^The Mina and Everard Goodman Faculty of Life Sciences, Bar-Ilan University, Ramat-Gan 52900, Israel; ^2^Department of Immunology, Faculty of Medicine, Technion - Israel Institute of Technology, Haifa, Israel

**Keywords:** B cells, aging, immunosenescence, rejuvenation

## Abstract

Age-related alterations in the cellular composition of the B lineage are a major cause of the poor antibody response to vaccination and to infectious agents among the elderly population. The mechanisms leading to these changes are poorly understood. Recently, we have shown that these changes reflect, at least in part, homeostatic pressures imposed by long-lived B cells that accumulate with aging, and that aging in the B lineage can be reversed upon alteration of B cell homeostasis by depletion. Here we discuss homeostatic causes for B lineage immunosenescence, and the potential for its rejuvenation.

## INTRODUCTION

Health care progress, antibiotics, vaccination and improving life standards caused a dramatic increase in lifespan in recent decades. By 2050, almost 40% of the European and US population is predicted to be >60 years old [1]. This development raises global public and scientific interest in studying age-related diseases and in developing strategies to improve the elderly quality of life. A major age-related health issue is the increasing prevalence and severity of infectious diseases, which are the fourth most common cause of death in the elderly [2]. Vaccination has only limited success since aging is also associated with poor protective antibody response to vaccines. For example, seroprotection against influenza virus strains is only 29-46% in persons aged ≥75 years and 41%-58% in persons 60-74 years old [3]. Clinical attempts to enhance vaccination efficacy in the elderly based on improving antigen delivery [3-5] have yielded limited success, and other attempts to regenerate the immune system in aging through dietary intake yielded very questionable benefits [6]. Such insufficient progress justifies development of new innovative approaches to enhance immunity in aging. However, to facilitate this, better understanding of immunosenescence mechanisms is necessary. In the present perspective we discuss our recent findings in reversing B cell aging and their possible implications.

### Immunosenescence of the B lineage

The poor ability to mount protective antibody responses in aging is primarily the outcome of dramatic alterations in the composition of B cell compartments, and the inefficient functional responsiveness of the peripheral pool. In mice, follicular B cell number drops with age, and the relative proportion of the antigen-experienced fraction increases [7,8]. Thus, the diverse, young-like peripheral repertoire gradually becomes limited in aging [9-11], thereby lacking the naïve cells that are able to recognize and respond to new antigens. Nevertheless, the total number of B cells in aged mice remains unchanged [8,12].

Age-associated changes in the composition of peripheral B cells reflect both increased B cell longevity and decreased B cell generation in the bone marrow (BM) [12]. Reduced B cell generation in the aged BM is due to many reasons, including reduced B cell progenitor frequencies [13] and proliferative potential [14], decreased IL-7 production [15] and impaired V-DJ rearrangement [16]. The latter appears to result not from a reduction in recombination-activating gene expression but from changes in the expression of the E2A-encoded, E12 and E47 basic helix-loop-helix proteins that bind the IgH promoter [17]. In addition, defects in hematopoietic stem cells (HSCs) that influence B lymphopoiesis have been described in aging. These include defects in telomere maintenance [18-20]; epigenetic modifications [21]; skewing of lineage potential from lymphopoiesis towards myelopoiesis [22-24]; altered development in the BM [25] and failure to generate a naïve young-like B cell repertoire [26].

In the periphery, the extended survival of memory cells, homeostatic proliferation and clonal expansion contribute to the limited repertoire and the poor antibody response in aging [27-30]. Additional intrinsic defects found in the response of B cells from aged mice or humans include decreased CSR and expression of activation induced cytidine deaminase (AID) and E47 [31], reduced activation and expression of phosphortyrosine kinases and protein kinase C [32], decreased expansion of B cells in response to antigen [33], and reduced number and volume of germinal centers (GCs) [34]. In collaboration with the Dunn-Walters group [35], we studied Ig genes from GC B cells to elucidate which factors in the affinity maturation process change with age. Age-related changes in the pattern of hypermutation were seen, although the analysis of variable region heavy chain (VH) genes and their lineage trees shows that an alteration in the mechanism of somatic hypermutation is unlikely. The changes are due to founder cell effects and/or the process of selection. Striking tissue-specific differences were seen. All measurements indicated that selection of Ig genes may decrease in Peyer's patch GC but increase in splenic GC with age. These tissue-specific differences highlight the importance of considering the activation and effector sites when studying immune senescence. All these factors contribute to the reduced quality of the antibody response as measured by amount and affinity (reviewed in [29]).

Several attempts to reverse the immunosenescent phenotype of the B lineage based on modification of gene expression were performed. It has been shown that the expression of p16Ink4a and Arf, which are products of the Cdkn2a tumor suppressor locus that are involved in cell cycle regulation and used as molecular signatures of aging in rodent and human tissues [36], is significantly increased in all stages of B lymphopoiesis in old mice, particularly in pro-B and pre-B cells. Down-regulation of these genes, obtained by deletion of the Bmi-1 repressor or by shRNA, reverted the senescent phenotype in aged lymphoid progenitors and restored their proliferative defects [37]. A more recent study used a retroviral vector to restore E47 expression in splenic B cells from old mice. This treatment rescued some of the intrinsic age-related defects as revealed by up-regulation of AID and CSR and the improved B cell immune responses in senescent murine B cells.

### Homeostasis in B lymphopoiesis

Homeostatic pressures play critical role in hematopoiesis and fit well within the concept in which early cell lineages adapt their output to demand [38,39]. There are several examples where homeostatic regulation mechanisms do not substantially change with aging, such as red blood cell production during physiological and pathological changes [39], myeloid progenitor number and developmental potential [23, 40], proliferation and mobilization of stem cells in response to G-CSF [41], and the ability of BM precursors to generate well-functioning CD4 T cells in mice [42].

Homeostasis in the hematopoietic system is studied by Bromodeoxyuridine (BrdU)-labeling experiments to assess the degree of proliferation of newly generated cells. Over the years these studies evoked a large controversy on the production rates, division rates, and lifespans of mouse and human lymphocyte populations, leaving fundamental questions such as the maintenance of memory, the maintenance of a diverse naive lymphocyte repertoire, and the nature of homeostatic mechanisms largely unresolved [43]. Mathematical models have been used to extract parameters from labeling experiments for better interpretations of the results, which otherwise are very often misinterpreted. For example, we have used the combination of BrdU labeling and mathematical models to study the development and maturation of T cells [44, 45], B cells [25, 46] and NK cells (Elemans et al., manuscript in preparation). These studies led to the discoveries of feedbacks in T cell development [47], blind homeostasis in peripheral T cell populations [48], and phenotypic reflux in B cell development [25]. Using similar labeling experiments in young/adults mice, it has been calculated that out of the 1-2x107 immature B cells that are generated daily in the BM, only 3% enter the pool of mature B cells [49, 50]. Several experimental approaches to determine the life span of the mature B cells reached different estimates ranging from 3-4 days to several weeks or months [51-53]. A more recent study used conditional gene targeting to ablate B lymphopoiesis in the BM in order to determine the life span of B cells in the absence of B cell influx from the BM. This study concluded that naïve follicular B cells have half life of ~4.5 months [54]. Thus, in young animals, B cell output from the BM and the size of the peripheral compartment are regulated by homeostatic pressures and physiological adjustments that are set by competition for trophic resources such as BAFF [27] and for survival niches [55]. Once these resources become limited, steady state B cell numbers are achieved and maintained by means of equal production and loss rates [27]. However, the attempts to determine whether B lymphopoiesis is regulated by homeostatic pressures imposed by the peripheral B cell compartment have reached to contradicting conclusions. Thus, Park and Osmond [56,57] and Cancro and Allman [58] demonstrated homeostatic regulation, whereas Freitas and colleagues [59] suggested that B cell production in the BM is autonomously regulated.

The above conclusions, however, may not be applicable to the B lineage in aging, where it undergoes dramatic alterations in the cellular composition [8,11,13]. Early studies have shown that BM or purified HSCs from old mice failed to reconstitute the peripheral B cell compartment upon short-term of B cell ablation by cyclophosphamide or in BM chimeras [8,26,60], thereby supporting the argument that age-related changes in B lymphopoiesis are not the consequence of homeostatic regulations. More recent studies, however, proposed that HSCs in aging subjects have a different clonal composition with decreased lymphoid-biased HSCs [24,61], which could explain the inefficient B lineage reconstitution in the above studies. The finding that an increasing dose of old HSCs for reconstitution partially restores age-associated changes in the BM and in the periphery [26] supports this hypothesis.

Using the combination of BrdU labeling and mathematical modeling, we investigated the changes in B cell development during aging [14]. Our results point at a reduction of pro-B/pre-B proliferative capacity after passing the first rearrangement checkpoint, similar to what we have found in T cells [62]. In addition, they suggest a possible reduction of the number or quality of survival niches for the developing B lymphocytes, as the carrying capacity of the pre-B cell compartment was found to be significantly reduced in aging [13,40]. This effect may be related to the accumulation of recirculating B cells in the bone marrow with aging, as we have also found that the number of “static” (non-dividing) IgM+ B cells in the bone marrow increases with age, and thus effectively reduces even further the space available for the developing B cells.

### Reactivation of B lymphopoiesis in aging

In an attempt to find whether suppression of B lymphopoiesis in aging reflects homeostatic pressures, we adopted two experimental approaches allowing us to alter B cell homeostasis in old mice via B cell depletion. In these experiments, we examined the two alternative possibilities: 1) B cell lymphopoiesis will remain suppressed, implying that suppression of B lymphopoiesis with aging is due to progressive and irreversible changes in HSCs, as suggested [23,26,60]; or 2) B cell lymphopoiesis will be reactivated, implying that the age-related suppression is due to homeostatic regulation that can be reversed. Our first approach was based on a genetic setting allowing the inducible ablation of the BAFF-receptor (BAFF-R) gene and the consequential deletion of peripheral B cells, since BAFF-BAFF-R signaling is critical for survival of mature B cells [63]. Our second approach was based on antibody-mediated B cell depletion in wild-type mice, which is similar to the approach used in lymphoma and rheumatoid arthritis patients treated with rituximab [64]. The outcome of both approaches is the establishment of long-term, chronic B cell deficiency in vivo. The results we obtained conclusively indicated that suppression of B lymphopoiesis in aging reflects homeostatic pressures in the B lineage [65]. In both models we observed a revival of B lymphopoiesis after depletion, as reflected by both frequencies and absolute numbers of pro-, pre- and immature B cells in the BM of the old-treated mice. We further addressed whether these homeostatic changes are sensed by earlier BM progenitors. Indeed, we found that upon chronic B cell deficiency, frequencies of multipotent primitive progenitors (MPPs) and common lymphoid progenitors (CLPs) were significantly increased, thereby to support the reactivation of B lymphopoiesis. The expansion of these progenitors argues that the reported age-related changes in HSCs must be reversible. There are several possible explanations for reversing the age-related defects in HSCs, as follows. 1) The acquisition of defects in HSCs is regulated by the peripheral demands for B-cell. For example, a decreased demand for B cell production in aged mice due the accumulation of long-lived cells may lead to selection of slow cycling HSCs, whereas a chronic demand for B cells enforces continuous B cell production and this may select for rapid cycling HSCs. 2) HSCs are capable of clearing the accumulated defects to reverse cellular senescence. Since environmental factors [19] and epigenetic modifications [21] affect the entrance into cellular senescence, these changes may be reversible. Studies showing that aged skeletal stem cells exposed to factors present in young serum restore their proliferation and regenerative capacity [66] support this possibility. 3) A low frequency of unmutated HSCs exists in the old BM and are selected to expand and differentiate into B lineage cells upon B cell depletion. This implies that reactivation of B lymphopoiesis does not occur rapidly to allow expansion of lymphoid progenitors, thereby explaining the failure of BM cells from old mice to rapidly reconstitute the B lineage in BM chimera or after lymphoablation [26,60]. Moreover, this possibility is supported by studies proposing that age-related alterations in the B lineage reflect changes in the clonal composition of the HSCs [24] or dictated by clonal expansion of functionally distinct HSC population [61], and the identification of a subpopulation of HSCs in the BM of old mice that retains an equivalent potential to differentiate into lymphoid and myeloid lineages [61].

The depletion of B cells and reactivation of B lymphopoiesis has rejuvenated the peripheral compartment. We showed that the long-lived antigen-experienced B cell compartments had been replaced by newly generated cells. These processes had also restored the “young-like” peripheral repertoire, but this has only been demonstrated using a repertoire-reporting immunoglobulin transgenic mouse model. Thus, further repertoire studies in normal old mice are necessary to validate this. However, the ability to rejuvenate the peripheral compartment by depletion of B cells is supported by studies showing that in patients treated with Rituximab, which depletes patients of B cells, the reconstituting B cells express a young phenotype [64] and a diverse repertoire [67]. A major question that arises in light of these findings is whether the rejuvenated B cells can mount enhanced immune response. Indeed, we found that upon administration of a foreign antigen, old mice that have rejuvenated their peripheral B cell compartment mounted a significantly enhanced antibody response relative to that of untreated age-matched mice. Yet, the response was still greatly reduced relative to the young controls, but this might be due to the age-related defects in the T lineage and in innate immune cells, which had not been replaced.

### Conclusions and future directions

**Figure 1.**
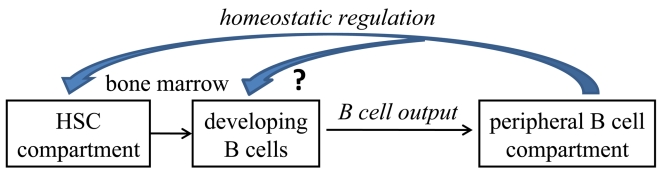
Based on our results we hypothesize that homeostatic pressures regulate the cellular composition of stem and B lineage cell compartments in aging. We suggest that these homeostatic pressures are set by the long lived B cells accumulating in the periphery with aging to alter the stem cell compartment and to suppress B lymphopoiesis. Changing the homeostatic equilibrium by depletion of B cells in old mice, increases frequencies of lymphoid-committed stem cells to reactivate B lymphopoiesis and to increase B cell output from the BM to the periphery. The nature of these cross-talk homeostasis mechanisms is yet to be defined, but our results suggest that they are sensed by the stem cell compartments.

The major conclusion of our study is that age-related alterations in the B lineage are reversible and mediated by homeostatic pressures imposed by the long-lived B cells accumulating in the periphery with age (figure 1). These observations are the foundations of new paradigms for enhancing immune responsiveness in aging, which may be translated in the future for clinical use. The nature of these homeostatic regulation mechanisms and the cross-talk between peripheral B cells and progenitor cell populations in the BM are yet to be identified. This will allow direct manipulation of B cell homeostasis by targeting the regulatory factor(s) rather than by depletion of B cells, to reactivate B lymphopoiesis and to enhance immune competence in the elderly.
